# Noninvasive, Targeted, and Non-Viral Ultrasound-Mediated GDNF-Plasmid Delivery for Treatment of Parkinson’s Disease

**DOI:** 10.1038/srep19579

**Published:** 2016-01-20

**Authors:** Ching-Hsiang Fan, Chien-Yu Ting, Chung‐Yin Lin, Hong-Lin Chan, Yuan-Chih Chang, You-Yin Chen, Hao-Li Liu, Chih-Kuang Yeh

**Affiliations:** 1 Department of Biomedical Engineering and Environmental Sciences, National Tsing Hua University, Hsinchu, 30013 Taiwan; 2 Medical Imaging Research Center, Institute for Radiological Research, Chang Gung University/Chang Gung Memorial Hospital, Taoyuan, 33302 Taiwan; 3 Department of Medical Science and Institute of Bioinformatics and Structural Biology, National Tsing Hua University, Hsinchu, 30013 Taiwan; 4 Institute of Cellular and Organismic Biology, Academia Sinica, Taipei, 11529 Taiwan; 5 Department of Biomedical Engineering, National Yang Ming University, Taipei, 11221 Taiwan; 6 Department of Electrical Engineering, Chang-Gung University, Taoyuan, 33302 Taiwan

## Abstract

Glial cell line-derived neurotrophic factor (GDNF) supports the growth and survival of dopaminergic neurons. CNS gene delivery currently relies on invasive intracerebral injection to transit the blood-brain barrier. Non-viral gene delivery via systematic transvascular route is an attractive alternative because it is non-invasive, but a high-yield and targeted gene-expressed method is still lacking. In this study, we propose a novel non-viral gene delivery approach to achieve targeted gene transfection. Cationic microbubbles as gene carriers were developed to allow the stable formation of a bubble-GDNF gene complex, and transcranial focused ultrasound (FUS) exposure concurrently interacting with the bubble-gene complex allowed transient gene permeation and induced local GDNF expression. We demonstrate that the focused ultrasound-triggered GDNFp-loaded cationic microbubbles platform can achieve non-viral targeted gene delivery via a noninvasive administration route, outperform intracerebral injection in terms of targeted GDNF delivery of high-titer GDNF genes, and has a neuroprotection effect in Parkinson’s disease (PD) animal models to successfully block PD syndrome progression and to restore behavioral function. This study explores the potential of using FUS and bubble-gene complexes to achieve noninvasive and targeted gene delivery for the treatment of neurodegenerative disease.

Parkinson’s disease (PD) is the second most common progressive neurodegenerative disorder and with over 70,000 new cases in the US each year. It is characterized by profound degeneration of mid-brain dopamine (DA) nigrostriatal neurons linked to serious motor symptoms. No definitive therapies are available that can attenuate disease progression, although some invasive deep brain stimulation can provide movement symptom control^1^,^2^. Drugs such as L-Dopa provide only symptomatic relief to patients who are hindered by drug resistance and progressive adverse side effects such as motor complications and dyskinesia, and gastrointestinal toxicity^3^. It has been shown that neurotrophic factor expression levels are decreased in PD patients^4^, and there is strong evidence that neurotrophic factor delivery can promote regeneration of DA neurons to relieve the syndromes of PD^5^,^6^,^7^. For example, glial cell line-derived neurotrophic factor (GDNF) is a potent agent for PD therapy due to its neuroprotective and neurotrophic effects^8^,^9^. Clinically, GDNF plasmid (GDNFp) gene delivery is feasible when using recombinant AAV vectors as a gene vector^10^,^11^.

Despite the potential for using GDNF to treat early stage PD, the molecular size of GDNF prevents penetration of the BBB, and viral and non-viral gene-carrying delivery requires local intracranial (IC) injection and infusion^12^,^13^,^14^. An alternative approach through systemic administration, such as with GDNFp macrophage-carrying system or GDNFp-loaded carrier, is also limited by off-target effects and the low therapeutic level achieved^15^,^16^. A novel approach for noninvasive and targeted GDNFp gene therapy is critically needed.

Focused ultrasound (FUS) sonication in conjunction with microbubbles (MBs) has been shown to transiently disrupt the blood-brain barrier (BBB) for noninvasive and targeted delivery of therapeutic substances^16^. MBs play a key role through interaction with FUS energy, which enhances microstreaming and acoustic cavitation, thus inducing mechanical stress to trigger transient tight-junctional morphological deformation^17^,^18^. Recently, it has been shown that FUS-induced BBB opening can facilitate viral gene delivery into the central nervous system (CNS)^19^, and also successfully deliver GDNF into the brain^20^,^21^. To address the gaps between conceptual feasibility and the use of FUS-triggered gene therapy, two concerns need to be addressed: (1) degradation with systemic administration and (2) sufficiently high gene expression at the targeted position.

For (1), GDNFp delivery by IV has excellent reticuloendothelial system (RES) escape capability concurrently with good biocompatibility. Given the design of a non-viral gene delivery system, a GDNFp-carrying carrier is needed to provide RES protection. For (2), the payload of the GDNFp cargo on the designed carrier should be large enough to achieve sufficient therapeutic effect and slow disease progression. MBs serving as a catalyst in FUS-BBB opening may play an additional role as a non-viral type GDNFp gene carrier. To achieve the above two conditions, the designed MB system should successfully trigger BBB-opening with FUS, and the MBs should be cationic to yield sufficiently high gene-carrying capability since the phosphate backbone of DNA is highly anionic^22^. Although cationic MBs (cMBs) have been previously attempted for enhanced FUS-mediated gene delivery in other organs^23^,^24^, to the best of our knowledge, there have not been any reports showing concurrent FUS-induced BBB opening and GDNFp gene delivery in CNS or for neurodegenerative disease treatment. Here, we propose a novel noninvasive, targeted, and non-viral GDNFp gene delivery system using FUS-induced BBB opening to treat PD (Fig. 1A). Our approach uses cMBs to yield high DNA payloads and we demonstrate that it can effectively reduce cell death in an animal model of PD.

## Results

### Cationic MBs have better GDNF plasmid binding ability than neutral MBs

**Figure 1:**
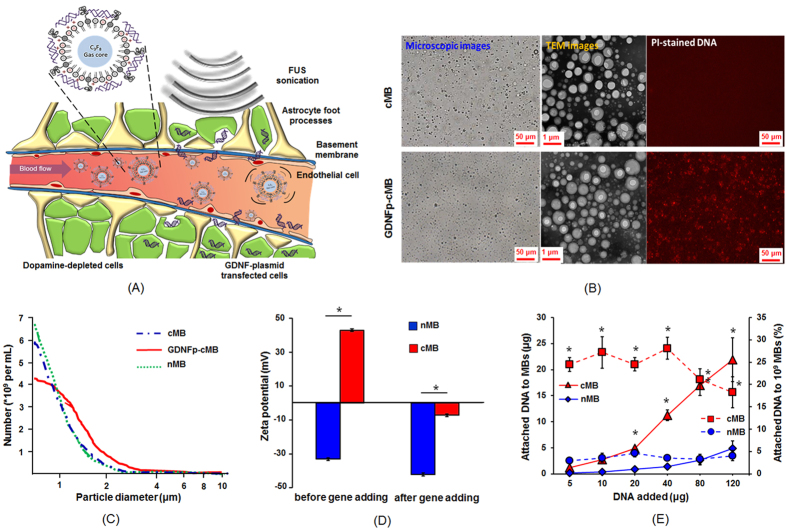
Concept and properties of GDNF-cMBs. (**A**) Schematic of GDNFp-cMBs and mechanism for controlled gene transfection of GDNFp-cMBs into brain triggered by FUS. (**B**) Left: Microscope bright-field images; middle: TEM images; right: PI staining image of cMBs and GDNFp-cMBs. (**C**) Size distributions of cMBs, GDNFp-cMBs and nMBs. (**D**) Zeta potential of nMB and cMB before and after adding GDNFp. (**E**) DNA loading efficiency of GDNFp onto nMB and cMB. The left axis was the amount of GDNFp bound onto MBs (solid line). The right axis was the GDNFp loaded efficiency onto MBs (dotted line). Single asterisk, *p* < *0.05*, versus nMBs. Data were analyzes by Student’s paired t-test presented as mean ± SEM (n = 6 per group).

The morphologic change in cMBs after conjugating with GDNFp was first observed through bright-field microscopy, cryo-TEM and fluorescent microscopy (Fig. 1B). The DNA binding of GDNFp-cMB was visually confirmed in fluorescence imaging via propidine iodide (PI) staining, showing a dense layer of spherical DNA (Fig. 1B). The mean diameter and concentration of the cMBs were 0.9 ± 0.4 μm and (3.3 ± 0.7) × 10^10^ MB/mL, and the mean positive zeta-potential was +39.5 ± 9.2 mV. The cMBs loaded with GDNFp had a slightly larger mean diameter and lower mean concentration (1.1 ± 0.1 μm and (2.5 ± 0.2) × 10^10^ MB/mL, respectively), but a negative mean zeta-potential (−7.1 ± 1.6 mV), indicating that the conjugation of GDNFp caused the MBs to shift from cationic to slightly anionic (see Fig. 1C,D). These results demonstrate the successful conjugation of GDNFp to the cMB lipid shell.

The DNA loading efficiency was based on the ratio of bound DNA plasmid to a bulk number of MBs (10^9^ as a reference). This was tested under different initial DNA amounts (range, 5–40 μg). The loading efficiency was significantly influenced by the zeta potential level, where the MBs in a cationic state showed significantly higher plasmid DNA loading efficiency (up to 26.1% ± 1.6%) due to better electrostatic interactions, whereas the binding efficiency was 7.1-fold higher than in the neutral MBs (nMBs; 3.7% ± 0.3%) (Fig. 1E). The plasmid DNA binding efficiency in cationic MBs was optimal at 40 μg of DNA during mixing. Further increasing plasmid DNA amount did not improve the loading efficiency.

### GDNFp-cMBs with FUS induced effective gene delivery and promoted cell differentiation *in vitro*

The gene transfection efficiency of FUS and GDNFp-cMBs was examined *in vitro* with PC-12 cells. GDNF triggers differentiation in PC-12 cells that is similar to that induced by neurotrophin nerve growth factor (NGF), and the effect can be evaluated though bright-field microscopy. Firstly, the successful transfection of gene-loaded cMBs into PC-12 cells was confirmed using FUS exposure with firefly luciferase gene-loaded cMBs (FLUCp-cMBs) via bioluminescence imaging (Fig. S1A,B). FLUCp gene expression started at 24 h after gene transfection and increased over time (Fig. S1C). In addition, increasing the concentration of FLUCp-cMBs enhanced the signal intensity of bioluminescence imaging, confirming the expression dependency with total payload (Fig. S1D).

After confirming the embedded gene on the cMBs can perform gene transfection following trigging of FUS, the bioactivity of GDNFp-cMB with FUS was examined through the following experiments. NGF supplied into PC-12 cells served as a positive control, and cell differentiation into neuronal phenotype was determined by neurite outgrowth (Fig. S2; 74.4 ± 19.2 μm; and 4.2 ± 0.8 μm in NGF-absence as negative control). GDNFp alone had a very limited effect on cell differentiation (8.6 ± 2.8 μm). FUS exposure alone slightly increased the neurite outgrowth (17.0 ± 4.0 μm). The effect of cell outgrowth was further enhanced when cMBs with FUS exposure was concurrently added with GDNFp (33.8 ± 7.8 μm). However, GDNFp-cMBs complexes triggered by FUS induced the most strong GDNFp gene transfection and expression (68.3 ± 24.5 μm), resulting in nearly 2- and 16-fold increases in neurite outgrowth, respectively, compared with the cMBs+FUS+GDNFp group and control group. The cell differentiation effect was almost equivalent to that of the NGF group. These results demonstrate the high gene delivery efficacy of the GDNFp-cMB and FUS system.

### GDNFp-cMBs and FUS exposure increase local BBB permeability and achieve local gene transfection *in vivo*

Next we investigated capability of employing GDNFp-cMBs complex triggered by FUS to open the BBB near the substantia nigra (SN) and striatum (Str) (acoustic pressure distribution of 1-MHz FUS exposure, Fig. S3; experimental setup; Fig. S4). 0.7-MPa FUS exposure induced sufficient BBB opening, as demonstrated by deep Evans blue (EB) staining in the local brain parenchyma that covered the entire Str and SN (Fig. S5A), but did not induce erythrocyte extravasations as the 1.0-MPa sonication one confirmed from hematoxylin and eosin (H&E) staining (Fig. S5B). We therefore selected 0.7 MPa as the FUS exposure level for subsequent *in vivo* gene transfection.

**Figure 2:**
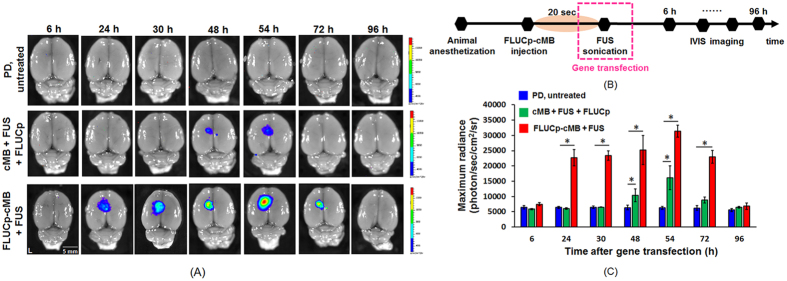
*Ex vivo* bioluminescent imaging after treatment. (**A**) *Ex vivo* bioluminescent imaging acquired 6, 24, 30, 48, 54, 72 and 96 h after treatment. Upper: control rat without gene transfection; middle: cMB+FUS+FLUCp group; bottom: FLUCp-cMB+FUS. (**B**) Timeline of bioluminescent imaging after gene transfection. (**C**) Time course of bioluminescent intensity. Single asterisk, *p* < *0.05*, versus PD untreated rat. Data were analyzes by one-way ANOVA (post hoc test: Dunnett; degrees of freedom: 6; F value: 9.7) and presented as mean ± SEM (n = 3 per group).

The efficacy of targeted transfection was evaluated via bioluminescence imaging of *ex vivo* brains. FUS-triggered FLUCp-cMBs resulted in FLUCp expression a 3.4-fold higher than concurrent MB/ FLUCp administration triggered by FUS ((3.3 ± 0.2) × 10^4^ vs. (1.6 ± 0.4) × 10^4^ photons/sec/cm^2^/sr; Fig. 2A–C), and also with a more profound rapid response as well as expression duration extension (24–72 h vs. 48–54 h). In a separate Western blot test, we also confirmed that only the FLUCp-cMBs system can produce sufficiently high Lucerfise gene expression (Fig. S6).

### GDNFp-cMBs and FUS treatment restore GDNF concentration of PD rat

**Figure 3:**
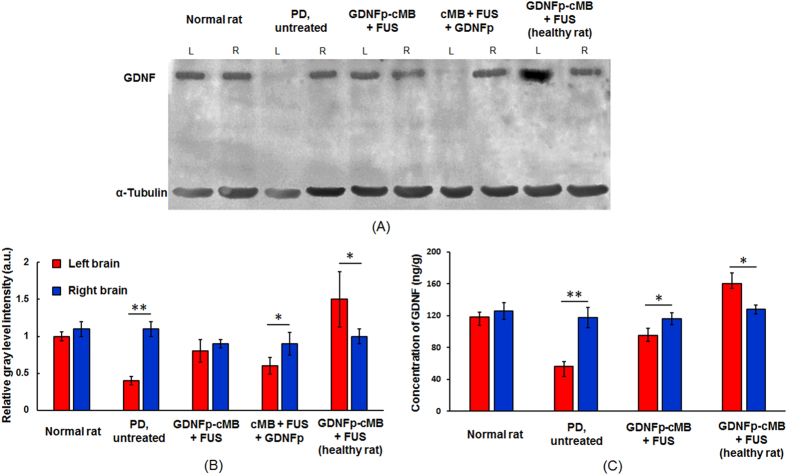
GDNF expression and concentration after treatment. (**A**) GDNF expression determined by Western blot. (**B**) Relative gray level intensity of Western blot. (**C**) GDNF concentration determined by ELISA. Single asterisk, *p* < *0.05*; double asterisk*, p* < *0.01*, versus right brain. Data were analyzes by student’s paired t-test and presented as mean ± SEM (n = 6 per group). All of the data were compared with right brain.

GDNFp expression *in vivo* was then analyzed via Western blot and ELISA. Western blot confirmed that the FUS-triggered GDNFp-cMBs system improved GDNF expression (Fig. 3A,B). In comparison to normal rats, the 6-OHDA injection site in PD animal presents a significantly reduced GDNF expression (33.3% compared to the contralateral site). It was noted that GDNFp-cMBs+FUS treatment provided the most profound GDNF expression recovery (85.6% compared to the contralateral site), which is superior than the cMB+FUS+GDNFp treatment (55.3% increase compared to the contralateral site). The enhanced GDNF expression via GDNFp-cMBs+FUS was also confirmed in normal rats, with the overly-expressed GDNF measured at the FUS-exposure hemisphere (167% increase compared to the contralateral site).

In ELISA analysis (Fig. 3C), the GDNF level in the toxin-injected brain was lower than in the contralateral (left brain: 56.7 ± 5.5 ng/g; right brain: 117.6 ± 12.9 ng/g). FUS-triggered GDNFp-cMBs gene delivery successfully restored the level of GDNF at the toxin-injected brain hemisphere (left brain: 95 ± 9.1 ng/g; right brain: 116.2 ± 7.3 ng/g), and was again confirmed in normal animal experiments where the treated brain hemisphere successfully restored the GDNF expression level (left brain: 160 ± 13.5 ng/g; right brain: 127.9 ± 5.3 ng/g). Similarly, FUS-triggered concurrent MBs/ GDNFp administration provided only minor recovery of GDNF expression (data not shown). These data show that our proposed technique can increase and restore secretion of GDNF *in vivo*.

### GDNFp-cMBs and FUS improve DA concentration and motor deficits in PD rats

**Figure 4:**
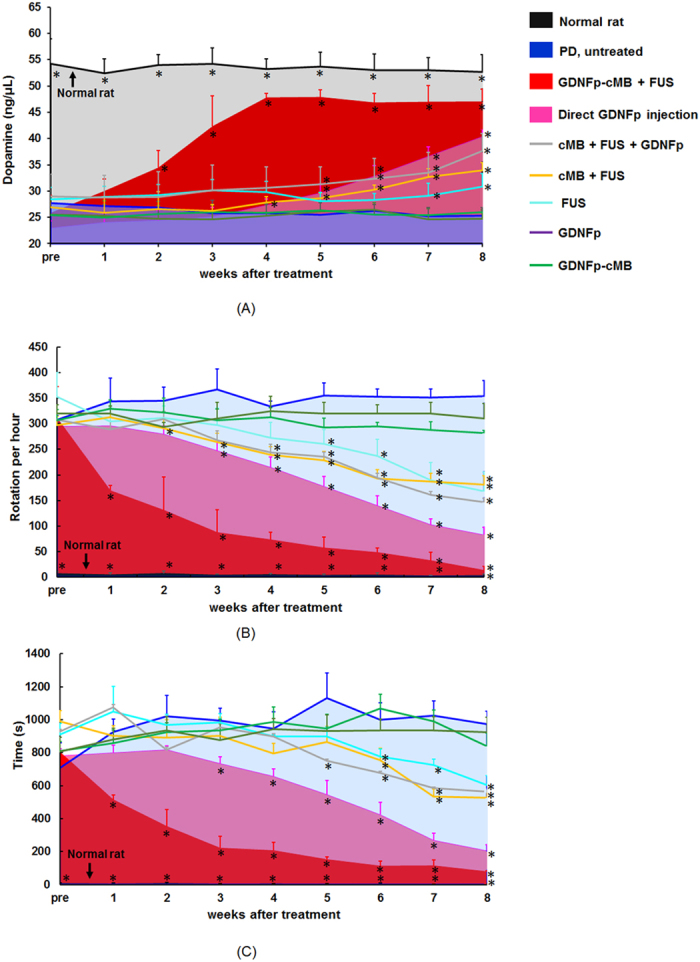
DA concentration and behavioral tests before and after treatment. (**A**) Change in DA concentration before and after treatment. (**B**) Change in apomorphine-induced rotational behavior before and after treatment. (**C**) Results of the bar test before and after treatment. Single asterisk, *p* < *0.05.* Data were analyzes by one-way ANOVA (post hoc test: Dunnett; degrees of freedom: 45; F value: 76, 149, 120.7) and presented as mean ± SEM (n = 6 per group). All of the data were compared with PD untreated rat.

We finally assessed the therapeutic effect of the FUS-triggered GDNFp-cMBs gene delivery for PD progression control. First, secreted DA was measured weekly to determine dopaminergic neurons status (Fig. S7; groups described in methods). Prior to treatment, the level of DA in PD rats was significantly lower than that in normal rats (27.7 ± 0.8 ng/μL vs. 54.2 ± 4.7 ng/μL), suggesting severe dopaminergic neuron loss caused by 6-OHDA (Fig. 4A). The extracellular DA levels were nearly restored by FUS-triggered GDNFp-cMBs gene delivered treatment, with a gradual recovery trend in the 4^th^ week (25.8 ± 3.4 ng/μL to 47.0 ± 2.4 ng/μL). Direct GDNFp injection and concurrent cMB/GDNFp administration + FUS exposure showed only partial restoration of DA levels, also with a response delay until the 8^th^ week (22.9 ± 1.4 ng/μL to 40.4 ± 1.4 ng/μL and 29.1 ± 4.2 ng/μL to 37.6 ± 3.3 ng/μL, respectively). Other groups failed to restore the DA levels at all.

Next, an apomorphine-induced rotational test was performed in parallel to evaluate motor asymmetry recovery (Fig. 4B). While the normal rats presented balanced motor function without abnormal rotation, the induced PD rats consistently showed abnormal rotation (initially with a rotation number of 307.9 ± 7.9 rotation/h, which increased to 354.3 ± 30.1 rotation/h at the 8^th^ week) as the PD-like syndrome developed. FUS-triggered GDNFp-cMB treatment produced the most profound abnormal rotation reduction (169.3 ± 10.2 rotation/h or 54.1% reduction at the first week treatment, eventually reaching a nearly fully normal level (13.2 ± 8.5 rotation/h or 95.8% rotation reduction)). Movie S1 demonstrates a clear motor function improvement caused by GDNFp-cMB with FUS in PD rats. Direct GDNFp injection also reduced the abnormal rotation when evaluated in the 8^th^ week (82.5 ± 14.8 rotation/h or 8.1% reduction), but there was no significant improvement in the 1^st^ week (330 rotation/h or 7.5% reduction). The treatment of cMB+FUS+GDNFp, cMB+FUS, and FUS alone also provides a rotation reduction (cMB+FUS+GDNFp: 146.8 ± 20.6 rotation/h; cMB+FUS: 167.6 ± 37.4 rotation/h; FUS alone: 181.3 ± 7.4 rotation/h).

Another motor-related behavioral bar test was used to measure the akinesia phenomenon of PD rats (Fig. 4C). In PD rats, the catalepsy time period ranged from 707.3 ± 53.5 s to 973.3 ± 27.7 s at week 8. The GDNFp-cMBs+FUS groups presented the most profound catalepsy duration (515.2 ± 28.7 s or 28.2% in the 1^st^ week treatment, reduced to 78.2 ± 6.14 s or 88.9% following the 8^th^ week treatment), suggesting repair of the motor-related deficit. Direct GDNFp injection also provided significant treatment efficiency (204.6 ± 6.0 s or 73.8% reduction at week 8), but did not have a therapeutic effect in the 1^st^ week (799.7 ± 45.4 s or only 2.4% reduction). Other groups showed no improvement or insignificant catalepsy reduction (GDNFp alone: 741.6 ± 48.8 s; GDNFp-cMB alone: 925.8 ± 18.1 s), implying very limited or no therapeutic effect (FUS alone: 605.2 ± 54.3 s; cMB+FUS: 564.4 ± 6.13 s; cMB+FUS+GDNFp: 564.4 ± 6.1 s). These results indicate that FUS-triggered GDNFp-cMB gene delivery is superior to the direct GDNFp injection in providing neuroprotection effect in PD rats both in terms of rapid curative response and normal behavioral restoration.

**Figure 5:**
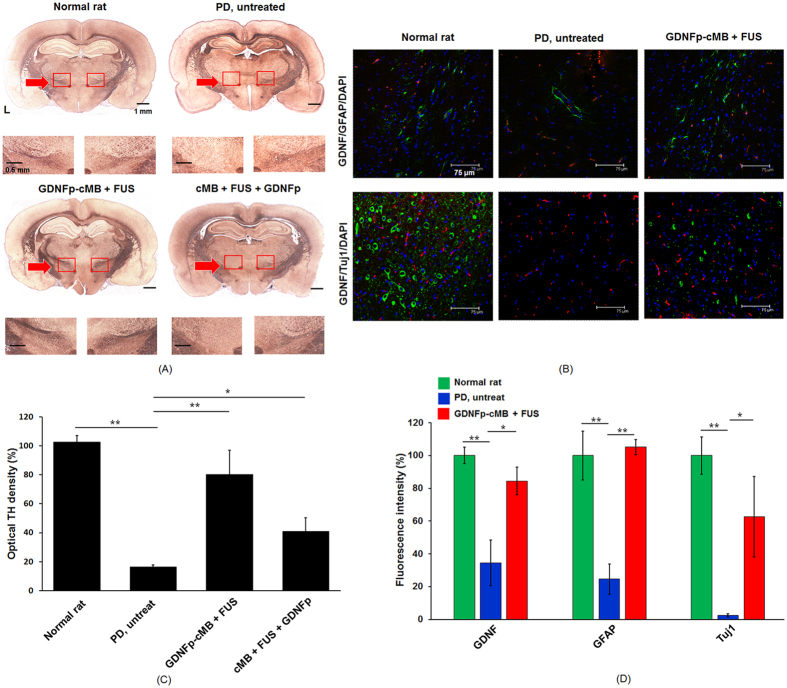
Immunohistochemical fluorescence staining. (**A**) Representative photomicrographs of SN sections immunostained for TH from normal rat, PD rat, GDNFp-cMB+FUS group, and cMB+FUS+GDNFp group. (**B**) IHC distribution of GDNF, GFAP, and Tuj1 expression in the sonication region after gene transduction. Transfected GDNF/GFAP/DAPI (top) and GDNF/Tuj1/DAPI (bottom) for the normal rat, PD rat, and PD GDNFp-cMB+FUS group, respectively. GDNF: red; Tuj1: green; GFAP: green; DAPI: blue. (**C**) The optical density of TH fibers in the lesioned hemisphere compared to the non-lesioned hemisphere. (**D**) The fluorescence intensity of GDNF, GFAP and Tuj1 in the lesioned site compared to the normal rat. Single asterisk, *p* < *0.05*; double asterisk, *p* < *0.01*. Data were analyzes by one-way ANOVA (post hoc test: Dunnett; degrees of freedom: 8, 6; F value: 46.9, 39.2) and presented as mean ± SEM (n = 6 per group). All of the data were compared with PD untreated rats.

To histologically confirm dopaminergic neuron recovery after GDNFp delivery, brain sections were stained via tyrosine hydroxylase (TH)-immunohistochemistry to observe the dopaminergic structures in the SN for potent neuroprotective and neurotrophic assessment (Fig. 5A). While TH-absence was observed at the 6-OHDA-injected site in untreated PD rats (16.4% compared with contralateral site), GDNFp-cMBs+FUS treatment successfully recovered dopaminergic neurons characterized by dense TH stains observed at the SN (80.2% compared with contralateral site), whereas recovery of the dopaminergic neurons was not observed in the cMB+FUS+GDNFp treatment group (41.0% compared with contralateral site) (Fig. 5C). Another analysis of counting TH-positive neurons within the SN after treatment also indicated that the density of DA neuron in GDNFp-cMBs+FUS group was higher than other group (GDNFp-CMB+FUS: 68.2 ± 18.7%; cMB+FUS+GDNFp: 41.4 ± 5.1%; PD: 18.8 ± 5.3%) (Fig. S8).

Immunohistochemical (IHC) fluorescence staining identified GDNF expression in CNS cells (Fig. 5B). Double-labeled IHC (GFAP for astrocytes (green) and DAPI for cell nuclei (blue)) was used to colocalize GDNF expression (red), and double-labeled IHC (Tuj1 for neurons (green), and DAPI for all cell nuclei (blue)) was conducted to see GDNF colocalization (red). Normal rats exhibited enriched GDNF expression (red) adjacent to astrocytes and neurons. With the injection of 6-OHDA, remarkable astrocyte and neuron death accompanied GDNF expression loss (GFAP: 14.6%; Tuj1: 3.4%; GDNF: 32.1% compared with normal rats). Treatment with GDNFp-cMBs and FUS eliminated the astrocyte/neuron loss (GFAP: 105.1%; Tuj1: 62.7% with normal rats). Notably, GDNFp-cMBs + FUS treatment showed that overexpression of GDNF (84.4% with normal rats), possibly due to secretion by glia cells, provided neuronal protection. This was confirmed by the enrichment of neural cells as shown by GDNF/GFAP/DAPI staining. These data indicate that FUS-triggered GDNFp-cMB gene delivery produce the targeted release of GDNFp, restoring GDNF secretion in PD brains.

## Discussion

In this study, we demonstrate that the FUS-triggered GDNFp-cMB platform can achieve non-viral targeted gene delivery via a noninvasive administration route, and show its superior growth-factor gene expression to present a neuroprotection effect in an animal model of PD. The designed cMBs are high-payload DNA-carrying vehicles that reduce the requirement for double IV administration of plasmid and MBs, and more importantly, have a superior gene transfection capability and *in vivo* circulation stability. We show that the proposed system significantly enhances targeted GDNFp gene delivery and expression, thereby improving the efficacy of gene therapy. The non-invasive approach via IV administration of gene-carrying cMB vehicles outperformed the traditional direct IC injection route with a surprisingly equivalent GDNFp titer.

MBs and FUS have been widely used to improve gene therapy *in vivo* and *in vitro* by enhancing the delivery of DNA to target cells. There are many possible mechanisms by which MB-induced cavitation modulates vessel permeability: (1) formation of transient and reversible pores on cell membranes (membrane sonoporation) that mediate intracellular macromolecule delivery^25^, (2) alteration of vascular endothelial integrity, permitting trans-vascular macromolecule transport between cells^26^, or (3) stimulation of endocytotic activity, promoting intracellular delivery^27^. However, there is no evidence that sonoporation-induced pores will transfect tissue beyond the vasculature. In our case, the area of gene transfection occurred in the vessels and brain parenchyma. Therefore, DNA may move from vessels to the extrasvascular side through intracellular gap junctions or active exocytosis. In addition, cavitation events and FUS radiation force may facilitate MB extravasation into the extrasvascular side, resulting in delivery beyond the vasculature.

This study used a high-throughput procedure to synthesize DNA-loaded MBs complexes. The negatively-charged DNA phosphate backbone could be electrostatically attached to the positive charges on the cMBs shell (DNA loading efficiency of nMBs and cMB: 3.67 ± 0.59% vs. 23.96 ± 3.71%). Although our data confirmed the treatment capability of this carrier, the loading and delivery efficiency of genes by MBs can be improved by changing the DNA loading strategies onto the surface of MBs (such as non-viral vector or viral particle) or by modifying the MB structure^28^,^29^. Tailoring the size of the MBs influences DNA loading. However, recent studies have implied that larger MBs have a higher risk of inducing brain damage during BBB-opening^30^. Furthermore, using specific targeting ligands with the DNA-loaded MBs enables the accumulation of MBs in areas of interest, thus improving the association between MBs and the vascular endothelium, and potentially enhancing the effects of gene delivery.

Our cell experiment indicated that FUS exposure alone increased the neurite outgrowth fourfold compared with the control group (17.0 ± 4.0 μm v.s. 4.2 ± 0.8 μm). Possible mechanisms for this include: (1) ultrasound-medicated cellular NO release, affecting cell proliferation^31^; (2) FUS stimulated intracellular signal pathway activated cell differentiation^32^; and (3) FUS might upregulate neurotrophin-3 (NT-3), which is a regulator of neural development differentiation^33^. Future studies may explore in greater detail the mechanisms by which FUS enhances cell neural differentiation.

Previous studies have indicated that the duration of FUS-induced BBB opening increased with the applied parameters of FUS and MBs dose^34^,^35^. Our H&E staining results indicate the parameters of FUS used in this study are safe, thus the duration of BBB opening should be a few hours. Lin *et al.* also demonstrated that the gene expression peak occurred at 48 h following the BBB opening procedure and IV injection of gene-loaded liposome^16^. Taken together, we suggest that the increased radiance for up to 54 h after treatment in the cMB+FUS+GDNFp and GDNFp-MB+FUS was due to delayed protein expression.

Our animal data illustrated that the combination of GDNFp-cMB with FUS could result in successful treatment of PD. However, a slight improvement in PD was also observed in the FUS group as well as the cMB+FUS group, implying that FUS or cMB+FUS might additionally stimulate neurogenesis of DA neurons in the SN and Str. The mechanisms underlying induction of neurogenesis by FUS with MBs still need to be elucidated. Lin *et al.* have demonstrated that FUS treatment upregulates trophic factors that promote neurogenesis, such as brain-derived neurotrophic factor (BDNF), vascular endothelial growth factor (VEGF) and GDNF^36^. In addition, recent studies pointed out that FUS with MBs can stimulate neurogenesis within the hippocampus, thereby modulating pathologic abnormalities, plasticity, and behavior in a mouse model of Alzheimer’s disease^37^,^38^,^39^. Therefore, perhaps FUS sonication alone also provides good treatment efficacy for PD. Future studies are needed to better understand the mechanism of FUS-induced neuronal cell repair and to optimize the parameters of FUS for enhancing treatment outcome of PD.

The cMBs+FUS+GDNFp group showed that IV administration of GDNFp following BBB opening did not result in as profound a treatment efficiency as in the GDNFp-cMB+FUS group. Injected GDNFp via IV administration may be degraded by nucleases in the bloodstream, thus limiting the amount of active GDNFp that enters the brain parenchyma through the BBB-opened capillaries. The cationic coating of the cMBs protects the bound DNA against nucleases^22^,^23^,^24^. Binding of the DNA to the cMBs also ensures that the plasmid is adjacent to BBB-opened capillary cleft and therefore can maximally enhance plasmid penetration benefit by FUS-induced microstream and radiation force. Another possibility is that FUS-induced microjets enhance the sonoporation and phagocytosis that enable gene entry into adjacent neuron cells. Sonoporation typically induces small pores up to 100 nm by imploding MBs with a short lifetime in the millisecond range^25^. It is therefore hypothesized that gene plasmid conjugated to the MBs can enhance FUS-induced sonoporation and phagocytosis.

Several artificial methods have been successful in opening the BBB for drug or gene delivery into the brain tissue, including (1) directly trans-cranial injection of agents, (2) administration of hypertonic solutions, and (3) sonication FUS following MBs injection. These methods have potential side-effects. In (1), an invasive catheter must be placed in the brain, resulting in brain trauma^40^. Also, the drug can only penetrate into the brain tissue via diffusion, which makes it difficult to cover the whole lesion from the deposited site. In (2), an increase of brain fluid may lead to aphasia, neurological toxicity and hemiparesis^41^. This method not only induces a non-localized drug delivery but also promotes the penetration of toxic substances into the brain tissues. In (3), over-exciting of FUS or high dose of MBs may produce intracerebral hemorrhage and brain tissue damage^30^,^42^. In this study, the histological data indicated that our used FUS parameters opened the BBB without brain tissue damage.

There are some limitations to overcome prior to clinical applications of this approach: (1) the 6-OHDA-induced PD rats did not recapitulate all the pathologic abnormalities observed in PD clinical cases, therefore these experiments need to be performed in other PD models to further confirm the efficacy of the delivery system; (2) only GDNF was tested. Further studies are needed to assess the effects of administration of BDNF, antibodies, or neurturin, in combination with FUS to the brain; (3) only one-time FUS exposure was used. Repeated FUS treatment might further enhance the effect of brain-drug delivery, so multiple treatments could be another approach in future PD treatments.

In this study, we proposed a noninvasive, targeted gene delivery system based on the combination of cMB-gene plasmid complexes with FUS. The proposed system features a high gene-plasmid payload and circulation stability, high gene transfection/expression, and a rapid response in disease progression control. So far, we have only examined the therapeutic potential in one animal model of PD, but the results support the possibility of a new direction for noninvasive and targeted delivery of gene therapy for neurodegenerative diseases.

## Materials and Methods

### Preparations of gene-insert plasmids

Plasmid encoding glial cell line-derived neurotrophic factor (GDNFp, OriGene, MD, USA) or firefly luciferase gene (FLUCp, OriGene) driven by a cytomegalovirus (CMV) promoter were both used in this study. FLUCp, was used to evaluate FUS-mediated transfection efficiency *in vitro* and *in vivo* via bioluminescence imaging. Both plasmids were propagated in Escherichia coli, extracted and purified by a plasmid extraction kit (NucleoBond Xtra Maxi EF, Macherey-Nagel, Düren, Germany) according to the manufacturer’s guidelines. The concentration and purity were determined by measuring UV absorbance at 260/ 280 nm with a spectrophotometer (NanoDrop 2000, Thermo Fisher Scientific, IL, USA).

### Preparation of the GDNFp-cMBs and FLUCp-cMBs

The cMBs were fabricated by the thin-film hydration method. Dipalmitoyl-phosphatidyl-choline (DPPC), 1,2-distearoyl-sn-glycero-3-phosphoethanolamine-N-[methoxy(poly(ethyleneglycol))-2000] (DSPE-PEG2000) and 1,2-dipalmitoyl-3-trimethylammonium-propane (DPTAP) (Avanti Polar Lipids, AL, USA) were dissolved in chloroform at a molar ratio of 9 : 2 : 1, and drained via rotary evaporator to form a dried film. The film was then hydrated with 5 μL/mL glycerol-PBS. Mechanical shaking by agitator for 45 s gave rise to cMBs with a C_3_F_8_ gas core and a lipid shell. Then, cMBs were separated from unreacted lipids by centrifugation at 6,000 rpm (2000 g) for 3 min. GDNFp/ or FLUCp (500 μg) was mixed with 10^8^ cMBs, gently rotated for 30 min, and then centrifuged at 6,000 rpm (2000 g) for 1 min to separate unloaded GDNFp/ FLUCp from well-conjugated GDNFp-cMBs/ FLUCp-cMBs. The normal MBs (nMBs) were designed by DPPC and DSPE-PEG2000 for comparison.

### PD animal model

All animal experiments in this study were permitted by the Institutional Animal Care and Use Committee of National Tsing Hua University and adhered to the experimental animal care guideline (IACUC approval number: NTHU10156). PD syndromes were established in 135 rats by 6-hydroxydopamine (6-OHDA). The 6-OHDA solution (10 μg 6-OHDA dissolved in 4 μL of 0.9% NaCl and 0.02% ascorbic acid) was stereotactically IC injected into the medial forebrain bundle of the left brain (anterior-posterior: −4.3 mm from the bregma; lateral: 1.6 mm with respect to the midline and ventral 8.2 mm from skull) by a syringe pump with a flow rate of 0.1 μL/min. One week post-surgery, animals received apomorphine-induced rotation tests weekly to confirm the successful PD lesion (detailed description in SI). It was established that animals that rotate a total of ≥250 contralateral turns/hour had a maximal (e.g., ≥99%) depletion of striatal DA. Only animals that manifested this rotational behavior underwent subsequent treatment.

### *In vivo* experimental setup of FUS-triggered gene transfection

Before starting the experiment, animals were anesthetized by the intraperitoneally (IP) injection of a mixture of Zoletil and Rompun (30 mg/kg, Virbac Laboratory, France). A PE50 catheter was inserted into the jugular vein for intravenous (IV) administration of genes, MBs, and dyes. *In vivo* gene transfection was conducted by self-integrated ultrasound imaging-guided FUS system sonication^29^ and FLUCp-cMB/ GDNFp-cMB (Fig. S2). Briefly, a 25-MHz transducer (V324, Panametrics, MA, USA) for image guidance and the 1-MHz transducer (V302, Panametrics) for transmitting a sonication pulse were arranged by a special holder to fix the foci of the transducers at the same depth. The pulse of FUS was delivered precisely to the treatment area by ultrasound imaging guidance.

### Verification of FUS with GDNFp-cMB induced BBB-opening and brain damage

Six PD rats were used to assess successful FUS BBB-opening. Rats were exposed transcranially to 1-MHz FUS irradiation with the acoustic pressure ranging 0.4–1.0 MPa with an exposure duration of 90 s at Str and SN (5,000 cycles, 1-Hz PRF). Each rat received 0.14 mL of GDNFp-cMB diluted with 0.36 mL 0.9% normal saline. Sonication started 20s after injection of GDNFp-cMB. The BBB-opening area was verified by staining with EB dye (100 mg/kg) (Sigma-Aldrich, MO, USA) which was administered 10 min before FUS sonication. When finishing the experiment, the animals were sacrificed and perfused with 750 mL of 0.9% normal saline through the left ventricle. Brain tissues were collected, fixed in 10% formalin (Sigma-Aldrich), embedded in optimal-cutting-temperature compound (Tissue-Tek, Sakura, CA, USA) and stored at −50 °C. The frozen brain tissues were sliced into coronal sections. EB extravasation served as an index of BBB-opening, in which the blue-stained distribution is easily visible to the naked eye. Tissue sections (thickness of 15 μm) were then stained with H&E to detect the presence of erythrocyte extravasations, a major adverse effect when tissues experience ultrasound over-exposure. Histological evaluations were conducted under the light microscope by a person who was blinded to the ultrasound parameters but informed of which side of the brain had been exposed.

### Bioluminescence Quantification of Transfection in Animal

The activity and location of gene delivery were identified at 6, 24, 30, 48, 54, 72, and 96 h after treatment via FLUCp-cMB integrated with bioluminescence imaging (IVIS-200, Xenogen Corporation, Ca, USA). For comparison, 63 PD rats were divided into three groups: control group (*N* = 21), FLUCp-cMB + FUS group (*N* = 21) and cMB+FUS+FLUCp group (*N* = 21). Twenty seconds after FLUCp-cMB administration, FUS was applied transcranially with an acoustic pressure of 0.7 MPa (MI value: 0.7), cycle number of 5,000, PRF of 1 Hz, and duration of 90 sec. The FUS was delivered into the Str and SN in the left brain. Ten minutes prior to bioluminescence imaging, the rats were IP injected with D-luciferin (150 mg/kg; Biosynth AG, Staad, Switzerland) and the rat brains were removed and immediately placed in the IVIS system. The signal intensity of the acquired frames was quantified by Living Image 3.0 software (Caliper Life Sciences, MA, USA) to assess transgene expression activity.

### PD Animal Treatment Efficiency Evaluation

Fifty-four PD rats were randomly divided into nine groups: PD rats without treatment (PD untreated, *N* = 6), IV injection GDNFp alone (GDNFp, *N* = 6), PD rats with direct GDNFp IC injection (direct GDNFp injection; as positive benchmark, *N* = 6), PD rats with GDNFp-cMBs IV administration alone (GDNFp-cMB, *N* = 6), PD rats with FUS exposure alone (FUS, *N* = 6), cMB with FUS exposure (cMB+FUS, *N* = 6), PD rats with concurrent MB/GDNFp administration and FUS exposure (cMB+FUS+GDNFp, *N* = 6), and FUS-triggered GDNFp-cMBs gene delivery to treat PD rats (GDNFp-cMB+FUS, *N* = 6). Normal rats without PD (*N* = 6) were used as controls. Each rat received 0.14 mL of GDNFp-cMB diluted with 0.36 mL of 0.9% normal saline. Sonication started 20 s after injection of GDNFp-cMB. Rats were exposed transcranially to 1-MHz FUS irradiation at the Str and SN in the left brain (0.7 MPa, 90 section, 1 Hz PRF, 5,000 cycles). Apomorphine-induced rotations, climb test, and *in vivo* microdialysis were applied weekly to trace the motor asymmetry, akinesia phenomenon and DA levels for 8 weeks (detailed description in SI).

### Enzyme-linked immunosorbent assay-based measurements (ELISA) of GDNF

The brains were removed and homogenized with RIPA buffer (APOLLO, CA, USA) containing protease inhibitors at 54 h after GDNFp-cMB and FUS treatment. The solutions were then centrifuged at 13,000 g for 30 min at 4 °C. The supernatants of the centrifuged solution were collected and added into the 96-well immunoplate (SPL, Kyounggi-do, South Korea). GDNF was detected with chicken anti-GDNF antibodies (Thermo Fisher Scientific, MA, USA) and secondary goat anti-rabbit IgG-HRP (horseradish peroxidase) antibody (Thermo Fisher Scientific). The HRP-labeled secondary antibodies were visualized using the TMB peroxidase substrate (Bethyl Laboratories, TX, USA) and ELISA stopping solution (Bethyl Laboratories). These samples were measured at 450 nm on a fluorescence plate reader system (Tecan Infinite M200, Tecan Trading AG, Switzerland) to quantify the concentration of GDNF.

### Immunohistochemical Fluorescence Staining

Tissue sections were stained overnight at 4 °C with the following primary antibodies: chicken anti-GDNF, mouse anti-GFAP (glial fibrillary acidic protein, Dako, CA, USA), and mouse anti-Tuj1 (neuron-specific class III beta-tubulin, Covance, NJ, USA). After rinsing in PBS, the sections were incubated in secondary antibody with goat anti-chicken fluorescence 594 (for GDNF; Molecular Probes, NY, USA) or with donkey anti-mouse fluorescence 488 (for GFAP or Tuj1; Molecular Probes) for 1 h at room temperature. After rinsing in PBS, coverslips were placed on slides with an anti-fade reagent with the nuclear marker DAPI (4′,6-diamidino-2-phenylinodole, Calbiochem, CA, USA). Finally, the sections were imaged by a confocal microscope (Leica Microsystems, Wetzlar, Germany). Counts were multiplied by the series interval to estimate the total number of quantified cells in the stained section with ImageJ. The intensity values for the GDNF, GFAP and Tuj1 on the ipsilateral side were expressed as a percentage of the normal rat, which was set as 100%.

### TH immunohistochemistry

The restoration of DA neurons in PD rats following treatment was confirmed by TH immunohistochemical staining. Eight weeks after the treatment, the brains of the rats were removed and post-fixed. Serial coronal sections of the brain (30 μm thickness) were obtained and incubated overnight with a monoclonal rat anti-TH IgG (Millipore Bioscience Research Reagents, MA, USA). After rinsing in PBS, the sections were incubated with secondary goat anti-rabbit IgG-HRP antibody for 1 h at room temperature. The activity of HRP was visualized and counted by DAB substrate (Thermo Fisher Scientific) by optical microscope. The optical density of the TH+ dopaminergic structures was measured by ImageJ. The microscope images of both SN were read as gray level and integrated. The intensity values for the SN on the ipsilateral side were expressed as a percentage of the contralateral non-lesioned side, which was set as 100%.

### Statistical analysis

All statistical evaluations including GDNF concentration, luciferase activity, DA concentration, score of behavior tests and number DA neurons were performed using one-way ANOVA. The behavioral assessment data were analyzed using the repeated measures two-way analysis of variance (ANOVA) followed by a post hoc test (Dunnett) for multiple comparisons or student’s t-test. The level of statistical significance was set at *p* value < *0.05*. All data are expressed as mean ± standard error of mean (SEM).

## Additional Information

**How to cite this article**: Fan, C.-H. *et al.* Noninvasive, Targeted, and Non-Viral Ultrasound-Mediated GDNF-Plasmid Delivery for Treatment of Parkinson’s Disease. *Sci. Rep.* **6**, 19579; doi: 10.1038/srep19579 (2016).

## Supplementary Material

Supplementary Movie S1

Supplementary Information
