# Supplementary material for: Noninvasive, Targeted, and Non-Viral Ultrasound-Mediated GDNF-Plasmid Delivery for Treatment of Parkinson’s Disease
Source: Sci Rep. 2016 Jan 20;6:19579. doi: 10.1038/srep19579 (PMC4726227; doi:10.1038/srep19579)
Supplement: Supplementary Information [file srep19579-s2.pdf]

SUPPLEMENTARY INFORMATION

Noninvasive, Targeted, and Non-Viral Ultrasound-Mediated GDNF-Plasmid Delivery for Treatment of Parkinson's Disease

Ching-Hsiang Fan[1], Chien-Yu Ting[1], Chung-Yin Lin[2], Hong-Lin Chan[3], Yuan-Chih Chang[4], You-Yin Chen[5] , Hao-Li Liu[2,6] & Chih-Kuang Yeh[1]

[1] Department of Biomedical Engineering and Environmental Sciences, National Tsing Hua University, Hsinchu, 30013 Taiwan

[2] Medical Imaging Research Center, Institute for Radiological Research, Chang Gung University/Chang Gung Memorial Hospital, Taoyuan, 33302 Taiwan

[3] Department of Medical Science and Institute of Bioinformatics and Structural Biology, National Tsing Hua University, Hsinchu, 30013 Taiwan

[4] Institute of Cellular and Organismic Biology, Academia Sinica, Taipei,11529 Taiwan

[5] Department of Biomedical Engineering, National Yang Ming University, Taipei, 11221 Taiwan

[6] Department of Electrical Engineering, Chang-Gung University, Taoyuan, 33302 Taiwan

Corresponding authors:

E-mail: ckyeh@mx.nthu.edu.tw (C.-K. Yeh), and haoliliu@mail.cgu.edu.tw (H.-L. Liu).

Table of Contents

METHODS

I. Characterization of GDNFp-cMBs complex

II. *In vitro* bioactivity of GDNF release from GDNFp-cMB complex

III. Experimental setup of ultrasound-triggered plasmid release from plasmid loaded-cMB complex

IV. Cell bioluminescence experiment

V. GDNFp-induced cell differentiation experimental design

VI. *In vivo* behavioral analysis

VII. *In vivo* microdialysis analysis of DA concentrations

VIII. Western Blot Analysis

REFERENCES

FIGURES

Fig. S1. (A) Experimental setup of *in vitro* gene transfection by FLUCp-cMB/ GDNFp-cMB with FUS exposure. (B) Scheduled time line of experiments. (C) Bioluminescence images with different concentrations of FLUCp-cMB after treatment. (D) Bioluminescence intensities with different concentrations of FLUCp-cMB after treatment.

Fig. S2. (A) Representative PC-12 morphology observation *in vitro* among experimental groups. Bar: 50 μm. (B) Schedule time line of experiments. (C) The length of cell neurite outgrowth in each group.

Fig. S3. Pressure distributions of the 1-MHz FUS exposure (A) along the beam axis; (B) along the cross-sectional plane at focal depth.

Fig. S4. Experimental setup of *in vivo* gene transfection by plasmid-DNA loaded cMBs triggered release by FUS.

Fig. S5. (A) EB extravasation in brain tissue after applying GDNFp-cMB and FUS with 0.4-1.0 MPa (Top: top view of brain; middle/ bottom: brain section. Bar: 1 mm. (B) H&E staining of FUS-exposure brain with pressure of 0.7 MPa.

Fig. S6. Western blot analysis of the BBB-opened brain region and its luciferase protein expression 54 h after completion of transduction.

Fig. S7. (A) Schedule time line of *in vivo* PD treatment experiments. (B) Experimental setup of *in vivo* microdialysis and HPLC-ECD system for longitudinal tracing of intracelebral DA concentrations.

Fig. S8. Density of neurons within the SN after different treatments.

MOVIE

Movie S1. Apomorphine-induced rotation behavior before and after treatment for normal rat, PD rat and PD rat treated with GDNFp-cMB with FUS.

SUPPLEMENTARY METHODS

I. Characterization of GDNFp-cMBs complex

The synthesized natural MBs (nMBs), cationic MBs (cMBs), FLUCp-cMBs and GDNFp-cMBs were counted and sized using a Coulter counter equipped with a 30 μm sensor orifice (Multisizer 3, Beckman Coulter, Miami FL, USA) for the 0.7–20 μm range, and their zeta-potential was detected using dynamic light scattering (DLS; Nanosizer-S, Malvern, London, UK). To evaluate the DNA binding capability of cMBs, 5-120 μg GDNFp were mixed with 10^9 cMBs (total volume of 250 μL). The DNA loaded on cMBs was precipitated with 3M sodium acetate and 100% ethanol. After 15,000 rpm centrifugation, the DNA pellet was washed with 70% ethanol, resuspended with TE buffer, and quantified by spectrophotometer. The DNA loading efficiency was calculated using the following formula:

$$DNA\ loading\ efficiency\ (\%) = \frac{weight\ of\ GDNFp\ loaded\ on\ 10^9 cMBs}{total\ weight\ of\ GDNFp\ added\ in\ cMBs} \times 100\% \qquad \text{(Eqn. S1)}$$

The binding of DNA onto the lipid shell of cMBs was imaged via propidine iodide (PI) stain (Sigma-Aldrich, MO, USA). The structure of cMBs and GDNFp-cMBs were visualized by a cryogenic transmission electron microscope (cryo-TEM; Tecnai F20, Philips, USA). Four microliter of samples were loaded onto carbon film with copper grids (HC300-Cu, PELCO, CA, USA). The grids were then

blotted at 100% humidity at 4°C and plunge-frozen into liquid ethane. The grids were stored under liquid nitrogen and transferred to the TEM with a 70 μm objective aperture. The dose condition of exposure was ~20 e-/Å2. Images were collected by a 4k × 4k CCD camera (Gatan, CA, USA).

II. *In vitro* bioactivity of GDNF release from GDNFp-cMB complex

The GDNF bioactivity was assayed *in vitro* using PC-12 cell line. This cell line can be differentiated to a neuronal phenotype in the presence of GDNF, inducing neurite outgrowth, and can well mimic neuron activity interacted with GDNF[S1]. PC-12 cells were supplemented with 15% horse serum (Gibco, NY, USA), 2.5% foetal bovine serum and 1% penicillin/strepto-mycin (Gibco) and maintained in Dulbecco's modified Eagle's medium containing growth factor F12 (DMEM/F12) (Gibco) and at 37 °C in humidified air with 5% CO_2.

III. Experimental setup of ultrasound-triggered plasmid release from plasmid loaded-cMB complex

A single element spherical focusing transducer (1-MHz, diameter: 25.4 mm, focus length: 52.7 mm; V302, Panametrics, MA, USA) was used to test the gene release with gene loaded cMBs. The transducer was driven by a homemade

impedance matching circuit, cascading with a function generator (WW2571, Tabor electronics, Haifa, Israel) and a radio-frequency power amplifier (Model 150A100B, Amplifier Research, Hazerswoude-Dorp, Netherlands). The half-maximum pressure amplitude of the transducer was measured at 2.9 mm in diameter and 24.8 mm in length using a calibrated polyvinylidene-difluoride type hydrophone (Onda, CA, USA). To direct the beam of FUS into the cell dish, a removable water cone was rested between the 1-MHz FUS transducer and the cell dish. In order to avoid cell contamination an ultrasound-transparent polyurethane membrane was covered between the cell dish and the water cone.

IV. Cell bioluminescence experiment

The gene transfection capability resulting from gene-loaded cMBs and FUS was visualized by PC-12 cell via bioluminescence imaging. Cells were evenly cultured in a six-well tissue culture-treated plate (12-well Microtest™ Plate, BD Falcon™, CA, USA) until reaching a cell density of 3×10^5 cells/well in 1 mL of culture medium. FUS exposure was applied 10 min after the addition of FLUCp-cMB (20 μL; concentration: (1-10) × 10^6 MB/mL), and dish wells without added sample assisted as controls. The medium (containing genes) was removed after 2 h incubation and the cells were washed with PBS and re-suspended in culture medium. FUS exposure was

applied with an acoustic pressure of 0.4 MPa, cycle number of 1,000, sonication site of 4, duration of 90 sec, and PRF of 1 Hz.

Living-cell bioluminescence after treatment was obtained via the bioluminescence imaging (IVIS-200, Xenogen Corporation, CA, USA). Twenty-four and forty-eight hours after treatment, the culture medium was removed from the six-well plate and replaced with 300 μL per well pre-warmed complete medium with D-luciferin (300 μg/mL, Biosynth AG, Staad, Switzerland). Images were acquired after 10 min of incubation with luciferin at 37°C. Luciferase activity was then analyzed and quantitated via Living Image acquisition and analysis software (Caliper Life Sciences, MA, USA).

V. GDNFp-induced cell differentiation experimental design

FUS was applied on PC-12 cells cultured on a 6-well tissue culture-treated plate with an exposure parameter described above beginning at 10 min after the addition of NGF, GDNFp, and GDNFp-cMB (20 μL; concentration: 10×10^6 MB/mL); within each dish sample the gene quantities were all adjusted to be identical at 10 μg. Six experimental groups were conducted in parallel including: control, NGF alone as positive control (25 ng), GDNFp alone, FUS exposure alone, concurrent cMB/ GDNFp addition triggered by FUS, and GDNFp-cMB triggered by FUS. After 7 days

of cell-culturing, neurite outgrowth was observed by inverted optical microscopy (IX-71, Olympus, NY, USA). The length of the cell outgrowth in each group was analyzed via image analysis software (ImageJ).

VI. *In vivo* Behavioral analysis

Motor activity in rats was evaluated by two behavioral tests specific for different motor abilities prior to 1 week and 1-8 weeks after treatment: (1) Apomorphine-induced rotation. Apomorphine (Sigma-Aldrich) is a dopamine agonist which is extremely effective in measuring rotational asymmetry in unilateral lesioned animals[S2]. The rats were intraperitoneally injected with apomorphine (0.5 mg/kg) and placed in a white hemispheric plastic rotation bowl (40 cm wide at top and 20 cm deep). The total number of full turns in the direction ipsilateral to the lesion was counted for 60 min. (2) Bar test to measure the akinesia phenomenon of the PD rats[S3]. During the test, the rat was placed on a table, and both forepaws were placed on a horizontal bar (diameter 0.7 mm), which was suspended 9 cm above the table. Total time (in seconds) spent by each paw on the bars was recorded to assess the akinesia severity caused by 6-OHDA toxin intracerebral injection as well as neuroprotection efficacy following gene delivery treatment.

VII. *In vivo* microdialysis analysis of DA concentrations

To evaluate the variability of DA concentrations within rat brain before and after treatment, two commercially available microdialysis probes (CMA 12, CMA, Solna, Sweden) were stereotaxically implanted into the both substantia nigra of the brain (anterior-posterior: -5.6 mm from the bregma; lateral: ±1.6 mm with respect to the midline and ventral 9.0 mm from skull) weekly. During sampling, animals were fixed in a stereotaxic frame and remained anesthetized throughout the experimental period. The body temperature of each rat was maintained at 37°C using a heating pad throughout the procedure. Dialysate collection began 2 h after insertion of the probe to allow some recovery from potential tissue damage caused by probe insertion. The probes were perfused with saline by a microinjection pump (CMA 402, CMA) at a flow rate of 2 μL/min for 15 min. The dialysis samples were then analyzed by high performance liquid chromatography coupled with electrochemical detector (HPLC-ECD; Decade II SDC, Antec, Leyden, Netherlands).

Then, dialysis samples were analyzed via HPLC-ECD. Briefly, 30 μL of the collected sample was loaded via manual injection through an Alltima amino C18 column (Grace Alltech, Breda, Netherlands) with a mobile phase (containing 1.4 g of 1-heptane-sulfonic acid, 0.1 g of ethylenediaminetetra acetic acid, 4 mL of phosphoric acid, 3.5 mL of triethylamine, 95 mL of acetonitrile and 970 mL of distilled and

deionized water) and detected by ECD. Quantifications of DA were based on peak heights and retention times relative to known concentrations of standard solution.

VIII. Western Blot Analysis

The rat brains were removed and homogenized with RIPA buffer (APOLLO, San Diego, USA) containing protease inhibitors at 54 h after GDNFp-cMB and FUS treatment. The solutions were then centrifuged at 13,000 g for 30 min at 4°C. The supernatants of the centrifuged solution were collected and protein quantification was performed with coomassie protein assay reagent (BioRad Laboratories, CA, USA). Thirty micrograms of protein samples were diluted with a Laemmli sample buffer (pH 6.8, 50 mM Tris, 2% SDS (w/v), 10% (v/v) glycerol, 0.01% (w/v) bromophenol blue) and divided by 1D-SDSPAGE. Following electroblotting the separated proteins onto 0.45 μm Immobilon P membranes (Millipore), the membranes were blocked with 5% w/v skim milk with TBS-T (pH 8.0, 50 mM Tris, , 0.1% Tween-20 (v/v), and 150 mM NaCl) for 1 h. The blocked membranes were then incubated in a primary antibody solution (chicken polyclonal antibodies to GDNF) in TBS-T containing 0.02% (w/v) sodium azide for 2 h and detected with the secondary antibody (goat anti-chicken IgG-HRP). The levels of proteins were normalized to α-Tubulin with goat polyclonal antibodies to α-Tubulin (Thermo Fisher Scientific) to correct loading differences in

cellular lysates. Membranes were washed by TBS-T and the expression levels of immunoprobed proteins were visualized with an enhanced chemiluminescence method. Specific protein bands were quantitated by densitometry (GE Healthcare, MI, USA).

REFERENCES

S1. Garbayo, E., Ansorena, E., Lanciego, J.L., Aymerich, M.S. & Blanco-Prieto, M.J. Purification of bioactive glycosylated recombinant glial cell line-derived neurotrophic factor. *Int. J. Pharm.* **344**, 9–15 (2007).

S2. Kreitzer, A.C. & Malenka, R.C. Endocannabinoid-mediated rescue of striatal LTD and motor deficits in Parkinson's disease models. *Nature* **445**, 643–647 (2007).

S3. Fantin, M. Auberson, Y.P. & Morari, M. Differential effect of NR2A and NR2B subunit selective NMDA receptor antagonists on striato-pallidal neurons: relationship to motor response in the 6-hydroxydopamine model of parkinsonism. *J. Neurochem.* **106**, 957–968 (2008).

SUPPLEMENTARY FIGURES

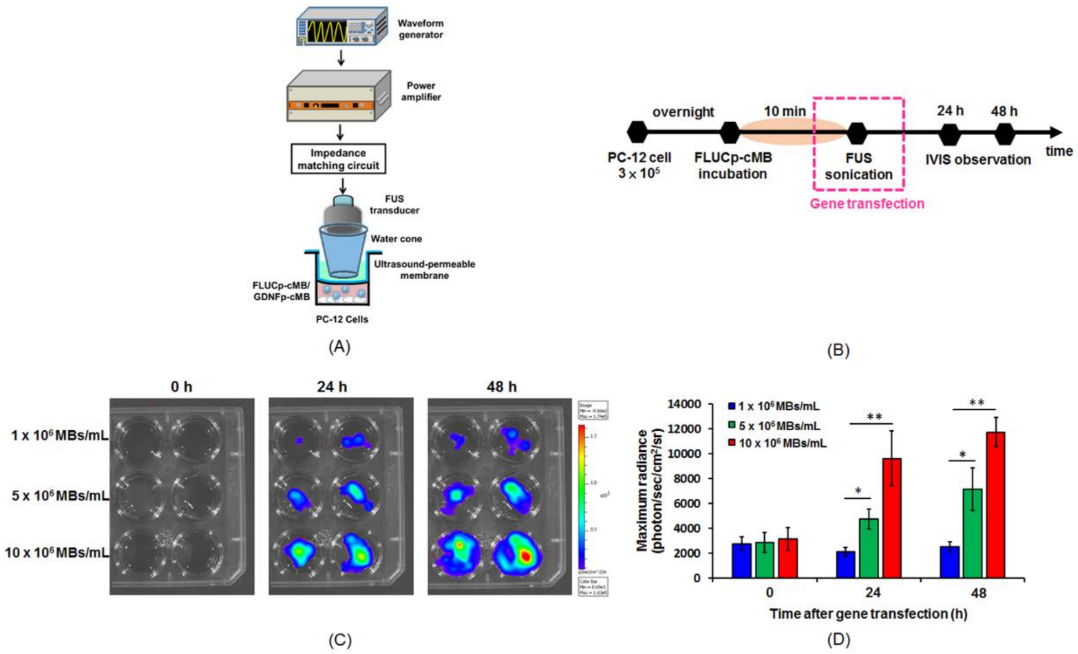

Fig. S1. (A) Experimental setup of *in vitro* gene transfection by FLUCp-cMB/ GDNFp-cMB with FUS exposure. (drawn by CHF) (B) Scheduled time line of experiments. (C) Bioluminescence images with different concentrations of FLUCp-cMB after treatment. (D) Bioluminescence intensities with different concentrations of FLUCp-cMB after treatment. Single asterisk, $p < 0.05$; double asterisk, $p < 0.01$, versus 1×10^6 MBs/mL group. Data were analyzes by one-way ANOVA (post hoc test: Dunnett; degrees of freedom: 9; F value: 132.9).

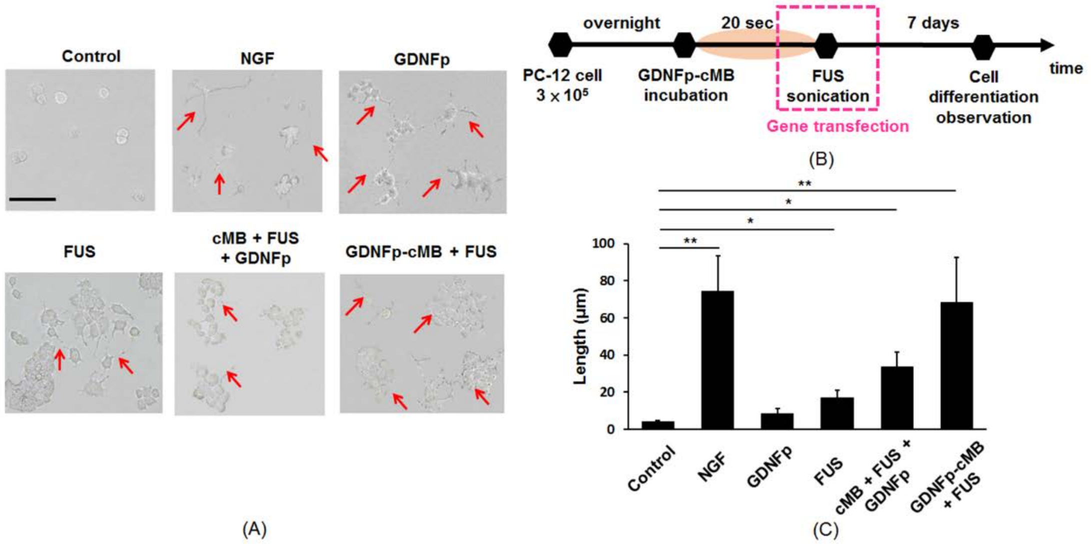

Fig. S2. (A) Representative PC-12 morphology observation *in vitro* among experimental groups. Bar: 50 μm. (B) Schedule time line of experiments. (C) The length of cell neurite outgrowth in each group. Single asterisk, $p < 0.05$; double asterisk, $p < 0.01$, versus control group. Data were analyzes by one-way ANOVA (post hoc test: Dunnett; degrees of freedom: 90; F value: 114.5) and presented as mean ± SEM (n = 15 per group).

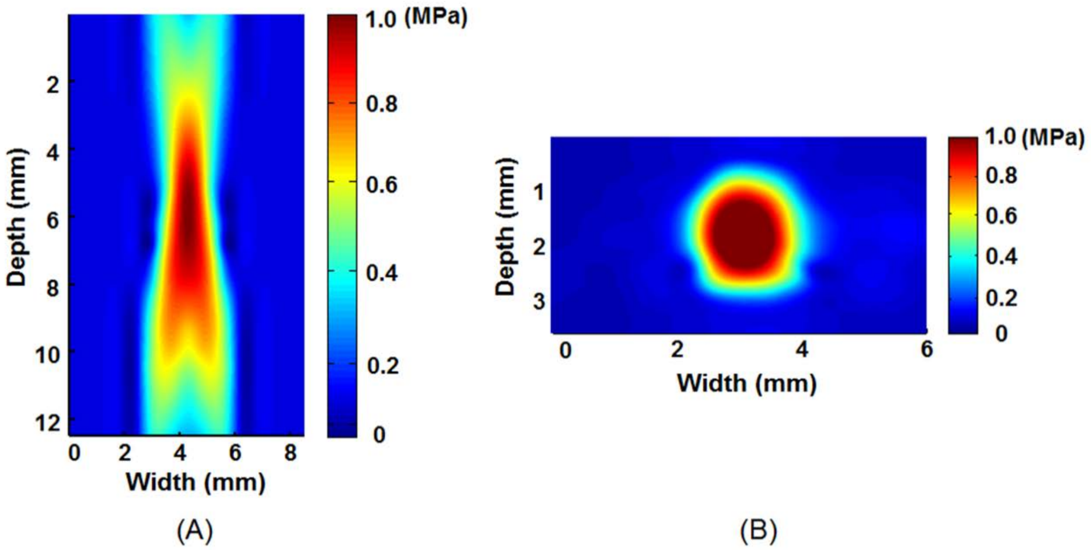

Fig. S3. Pressure distributions of the 1-MHz FUS exposure (A) along the beam axis; (B) along the cross-sectional plane at focal depth.

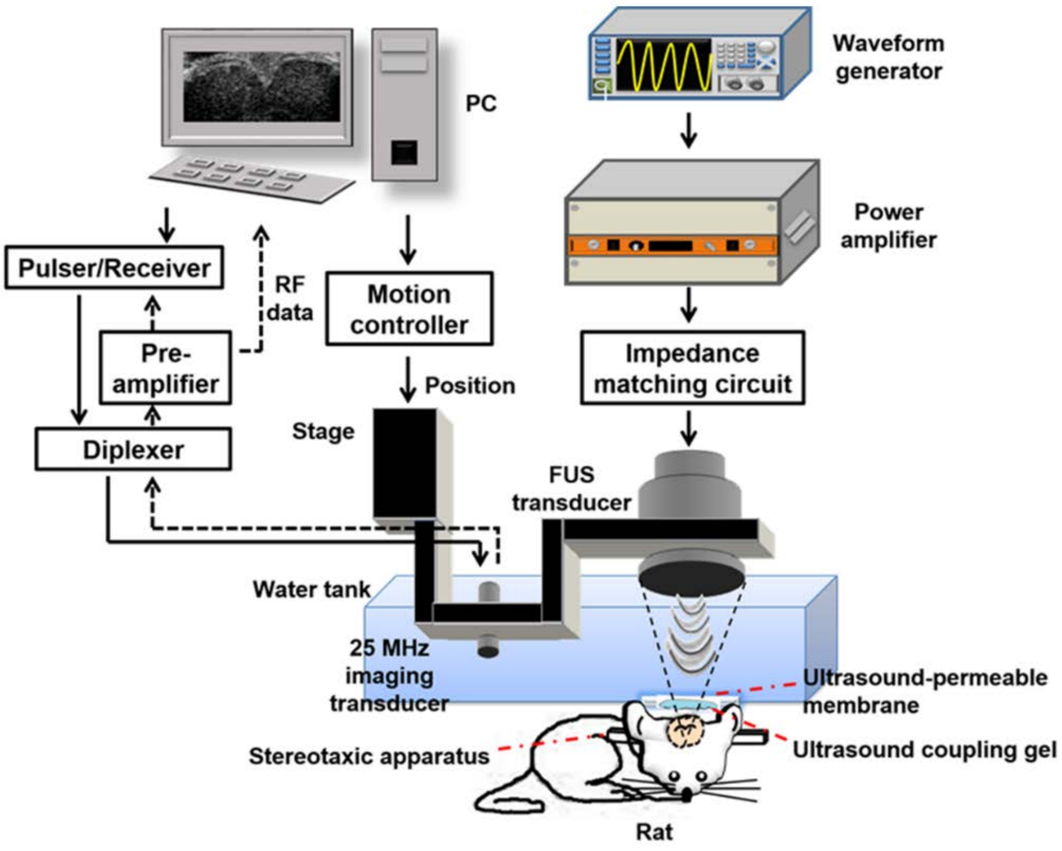

Fig. S4. Experimental setup of *in vivo* gene transfection by plasmid-DNA loaded cMBs triggered release by FUS.

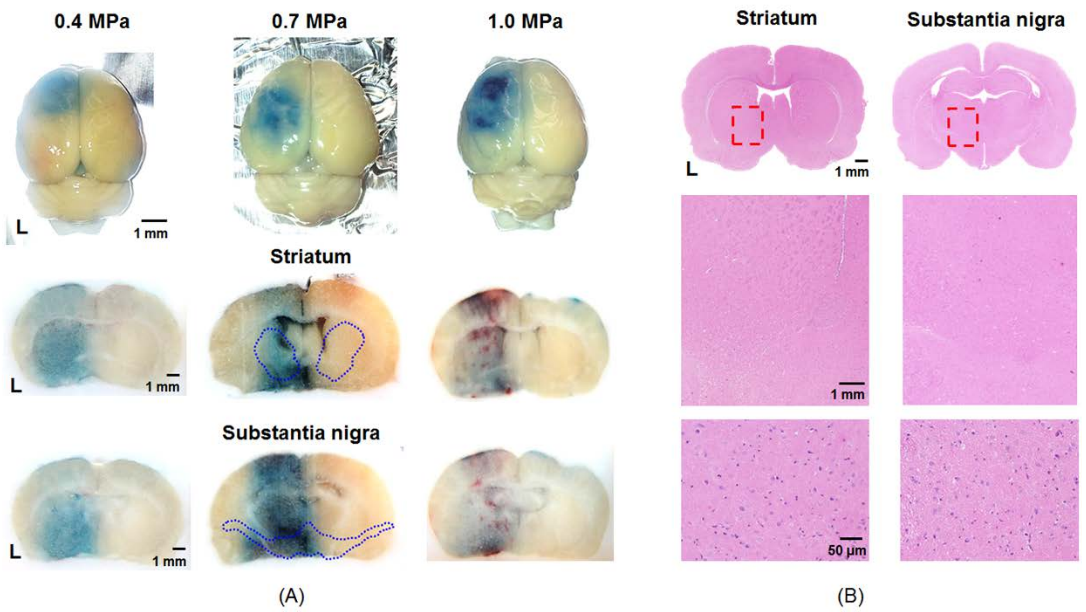

Fig. S5. (A) EB extravasation in brain tissue after applying GDNFp-cMB and FUS with 0.4-1.0 MPa (Top: top view of brain; middle/ bottom: brain section. Bar: 1 mm. (B) H&E staining of FUS-exposure brain with pressure of 0.7 MPa. Red rectangle: sonication area. Upper panels: 1×, bar: 1 mm; middle panels: 8×, bar: 1 mm; lower panels: 200×, bar: 50 μm.

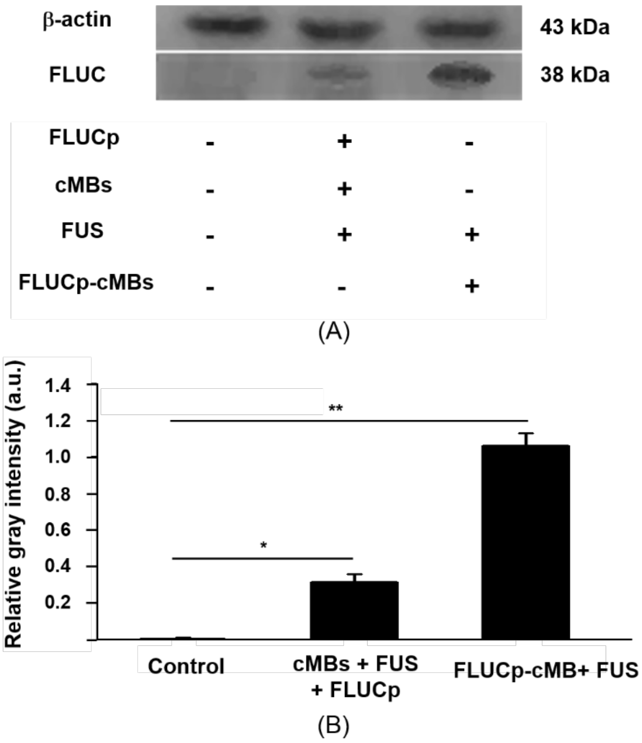

Fig. S6. Western blot analysis of BBB-opened brain region and its luciferase protein expression 54 h after transduction. (A) The bands exhibited a strong expression of luciferase after FLUCp-MBs with FUS sonication. Actin functioned as a control for protein loading. The following conditions are studied; Control, cMB+FUS+FLUCp group, and FLUCp-cMB+FUS group. Brain tissue lysates (10 μg/lane) were performed on a 10% SDS-PAGE and were immunoblotted with anti-luciferase pAb and anti-b-actin. (B) Relative gray level intensity of Western blot. Single asterisk, $p < 0.05$; double asterisk, $p < 0.01$, versus control group. Data were analyzes by one-way ANOVA (post hoc test: Dunnett; degrees of freedom: 6; F value: 20.6) and presented as mean ± SEM (n = 3 per group).

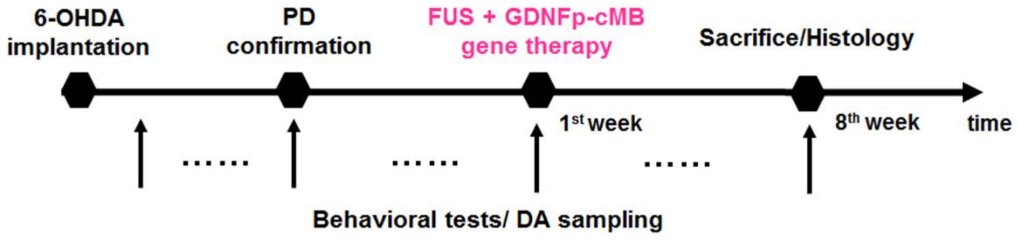

(A)

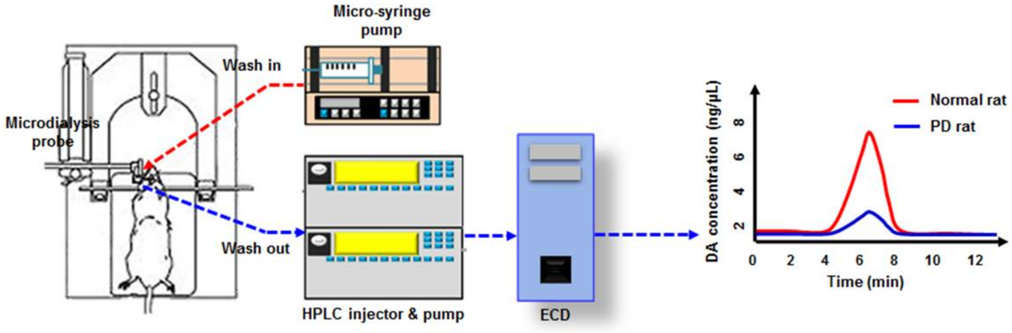

(B)

Fig. S7. (A) Schedule time line of *in vivo* PD treatment experiments. (B) Experimental setup of *in vivo* microdialysis and HPLC-ECD system for longitudinally tracing intracelebral DA concentration.

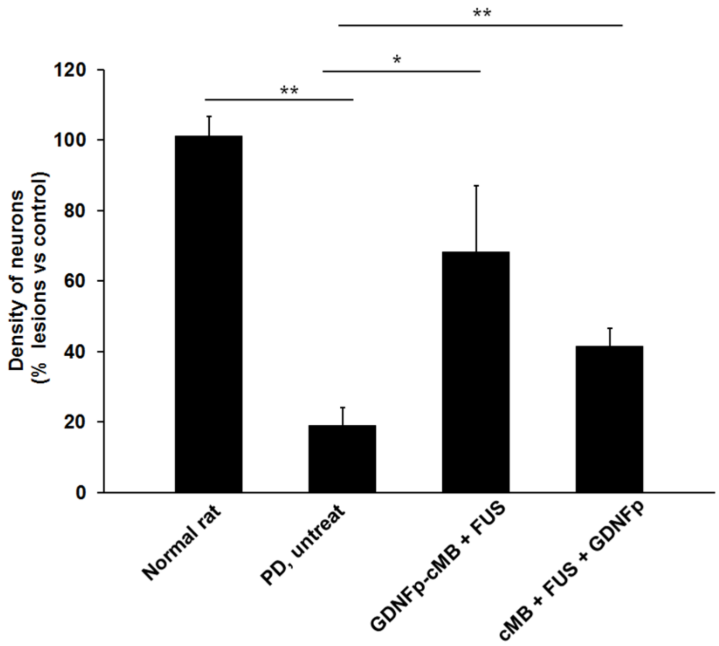

Figure S8. The density of TH-positive neurons within the SN after different treatments. The density of neurons within PD group was lower than normal group (PD: 18.8 ± 5.3%; normal: 101.8 ± 5.5%). After receiving the treatment of GDNFp-cMB and FUS, the neuron density of PD rats were obviously recovered (68.2 ± 18.7%). Single asterisk, $p < 0.05$; double asterisk, $p < 0.01$, versus PD untreated rat. Data were analyzes by one-way ANOVA (post hoc test: Dunnett; degrees of freedom: 80; F value: 120.3) and presented as mean ± SEM (n = 21 per group).

MOVIE

Movie S1. Apomorphine-induced rotation behavior before and after treatment from normal rat, PD rat and PD rat treated with GDNFp-cMB with FUS. (Sec 1-11: Before treatment; Sec 12-24: 8th week after treatment)
